# Respiratory pathogen analysis in pediatric inpatients unraveled the infection pattern of *Mycoplasma pneumoniae* post the COVID-19 pandemic

**DOI:** 10.3389/fpubh.2024.1437508

**Published:** 2024-10-09

**Authors:** Qihong Chen, Ruizhi Xu, Ying Gu, Jie Peng, Chiyuan Ma, Dubin Su, Shuai Liu, Dandan Ge, Yungang Yang, Wanshan Ning

**Affiliations:** ^1^Department of Pediatrics, the First Affiliated Hospital of Xiamen University, School of Medicine, Xiamen University, Xiamen, Fujian, China; ^2^Department of Pediatrics, Pediatric Key Laboratory of Xiamen, the First Affiliated Hospital of Xiamen University, Xiamen, Fujian, China; ^3^Institute for Clinical Medical Research, the First Affiliated Hospital of Xiamen University, School of Medicine, Xiamen University, Xiamen, Fujian, China

**Keywords:** COVID-19 pandemic, nonpharmaceutical interventions, respiratory pathogens, *Mycoplasma pneumoniae*, children

## Abstract

**Background:**

To counteract the COVID-19 pandemic, nonpharmaceutical interventions (NPIs) were implemented globally, exerting a profound influence on a wide spectrum of infectious diseases, encompassing respiratory tract infections (RTIs). Subsequent to the easing of NPIs, China experienced a significant outbreak of *Mycoplasma pneumoniae* (MP).

**Methods:**

Over a decade from 2015 to 2024, our study scrutinized 12 common infectious diseases among pediatric children. Etiologically diagnostic data and clinical outcome metrics of children with RTIs, tested for 13 pathogens, were analyzed to evaluate changes during and after the pandemic compared to pre-pandemic periods, with a notable emphasis on age profile and coinfection patterns of MP.

**Results:**

Among 57,471 hospitalized children, 23,178 were diagnosed with infectious diseases. Under NPIs, most respiratory infections declined compared to pre-pandemic levels, rebounding by 69.64% in 2023. While the infection rate of common respiratory pathogens decreased, cases of respiratory syncytial virus increased during the period of extensive NPI implementation. In 2023, pediatric intensive care unit durations for these pathogens increased, suggesting greater severity of illness compared to 2019. MP exhibited the highest infection rate (31.38% average), with a notable outbreak post-pandemic due to severity increase in <3 year olds and rise among older children. NPIs reduced MP coinfections and mitigated their severity, while exerting a significant influence on bacterial coinfections with MP over the span of 5 years, in contrast to their impact on viral pathogens.

**Conclusion:**

NPIs effectively curb transmission of respiratory infections by most pathogens, resulting in increased average age of MP infections and altered patterns of coinfection post-pandemic.

## Introduction

In early 2020, China implemented a wide range of public health and social measures (such as movement restrictions, mask mandates, social and physical distancing, environmental cleaning and disinfection) in response to the COVID-19 pandemic, which is also known as nonpharmaceutical interventions (NPIs). These measures not only successfully contained the spread of syndrome coronavirus 2 (SARS-CoV-2) but also significantly decreased the incidence of various community-acquired infectious diseases, including respiratory tract infection (RTI) which is primarily transmitted through aerial droplets, aerosols, and direct contacts (1–3).

RTIs may induce uncontrolled systemic inflammatory responses, leading to severe complications such as septic shock, acute respiratory distress syndrome, pneumonia, encephalitis, and multiple organ failure. They represent one of the primary causes of hospitalization and mortality in children. According to research statistics, in the year 2000 alone, approximately 1.9 million children globally died from RTIs, thereby imposing a significant burden on both families and society (4–6). Respiratory pathogens causing RTI primarily include respiratory syncytial virus (RSV), human adenovirus (HAdV), human rhinoviruses (HRV), influenza virus (IFV), *Bordetella pertussis* (causative agent of pertussis), *Haemophilus influenzae* (HI), and *Streptococcus pneumoniae* (SP). After NPI implementation during the COVID-19 pandemic, respiratory infections significantly declined, but their resurgence in children post-NPI withdrawal exceeded historical levels (7).

Among these pathogens, there has been also a resurgence of *Mycoplasma pneumoniae* (MP) in multiple countries occurring thereafter, especially China, triggering large-scale non-seasonal outbreaks (8–11). It serves as one of the most prevalent causative agents of community-acquired pneumonia in children, particularly infants and young children because of the lack of cross-protective antibodies generated from previous infectious diseases and high resistance to macrolide antibiotics (12, 13). Previous research has confirmed that MP can coinfect with other pathogens and the emergence of COVID-19 highlighted the severity and complexity of diseases caused by viral-bacterial coinfections again (14–16). However, existing studies have primarily focused on single-pathogen detection, with relatively few investigations into the mixed infection patterns dominated by MP in RTIs. Additionally, there is a lack of focus regarding the age-specific characteristics of MP infections in children before and after the implementation of NPIs.

Against the backdrop of a large-scale outbreak of MP in Chinese children post the COVID-19 pandemic, this work aims at investigating the overall infection situation among children (≤ 14 years old) hospitalized due to community-acquired infections in Xiamen, Fujian Province, China. We examined the epidemiologic characteristics of infectious diseases, especially RTIs from January 2015 to January 2024, and profile the changes of MP infection as a whole as well as by age group. Besides, we characterized its coinfection patterns with other respiratory pathogens. These findings are informative about the broad impact of NPIs post COVID-19 pandemic, especially on MP infection patterns, guiding the control of respiratory pathogens among children.

## Methods

### Study design and patient enrollment

A retrospective cohort study was conducted using data from the First Affiliated Hospital of Xiamen University. The First Affiliated Hospital of Xiamen University, the largest tertiary Grade-A comprehensive hospital in southwestern Fujian Province, serves the majority of children residing in the region.

This study enrolled all pediatric inpatients with infectious disease admitted from January 2015 to January 2024. To analyze trends in traditional respiratory pathogens, children infected with SARS-CoV-2 were excluded from the study. Diagnostics were recorded by each pediatrician using the International Classification of Disease 10th edition, which were detailed in Supplementary Table 1. Patients were divided into three groups according to their ages: 0–3 years, 3–6 years, and 6–14 years. This study followed the Strengthening the Reporting of Observational studies in Epidemiology (STROBE) guidelines.

### Study design and patient enrollment

Data were collected through review of medical records, including demographic information, timeline of symptom onset, hospitalization and pediatric intensive care unit (PICU) admission (if applicable), clinical presentations, laboratory test results, microbiological findings, and clinical outcomes. Trained clinical physicians inputted the standardized database.

To ensure the reliability of pathogen detection, all samples were meticulously collected by specialized nurses within 48 h of patient admission, followed by testing within 4–6 h of collection, while being stored in a controlled environment at 4°C throughout the process. Blood and sputum bacterial culture tests were used to detect SP, HI, *Staphylococcus aureus* (SA), *Klebsiella pneumoniae* (KLP), *Pseudomonas aeruginosa* (PA), *Legionella pneumophila* (LP), *Branhamella Catarrhalis*, Pertussis, and *Acinetobacter baumannii*. Urine can be utilized for the detection of bacteria such as *Escherichia coli*, KLP, PA, as well as the antigen of SP. Additionally, oral pharyngeal swabs were collected for viral pathogen detection, with total nucleic acids (both DNA and RNA) being extracted from these specimens. Subsequently, RT-PCR assays were conducted to identify IFV, RSV, human parainfluenza virus (HPIV), human metapneumovirus (HMPV), seasonal human coronavirus (HCoV), and HRV, while PCR assays were used to detect HAdV, human bocavirus (HBoV), MP, and Chlamydia. All tests followed the standard operating procedures established by the Chinese Center for Disease Control and Prevention, utilizing commercially available, mature pathogen detection kits (Shengxiang Biotechnology, Changsha, China) tailored for specific pathogens. Nucleic acid extraction and PCR/RT-PCR testing were performed in parallel with both positive and negative controls, and rigorous quality control measures were implemented. Each positive result was reviewed by at least one infectious disease physician. If testing positive for more than one pathogen, mixed infection is determined. We referred to cases that test positive for more than one pathogen as mixed infection.

### Infection rate

To better characterize the infection condition of various pathogens, we defined the infection rate (IR) as the number of cases infected by a specific pathogen divided by the total number of respiratory infections in that year.

### Regional interventions in Xiamen

Since January 26, 2020, Xiamen region initiated the promotion of NPIs to reduce the transmission of SARS-CoV-2. In June 2020, enhanced medical observation was implemented for incoming individuals to Xiamen. Subsequently, high-traffic areas such as entertainment venues, parks, and tourist attractions were gradually closed to curb the spread. Areas with confirmed COVID-19 cases were placed under lockdown. Notably, starting from March 21, 2020, school offline teaching activities were intermittently suspended based on the epidemic trend. Stringent epidemic containment policies were enforced from September 18, 2021, to August 25, 2022. Xiamen region lifted the epidemic containment policies on August 26, 2022.

### Data management and statistical analysis

All data are extracted from electronic medical records from January 2015 to January 2024, and the number of participants is counted bi-weekly. Descriptive statistics include the frequency (proportion) of categorical variables and the interquartile range (IQR) and median of continuous variables. To evaluate the impact of NPIs, we calculated the frequency and percentage of infectious diseases and pathogen diagnoses from January 2019 to January 2024. Using the 2019 data as the baseline, we quantified the changes from 2020 to January 2024 using a binary logistic regression model to analyze the trends. Statistical analysis was performed using STATA 15 (StataCorp 2015, College Station, TX) and R 4.3.2, with the significance level for assessing the relationships between variables set at *p* value <0.05.

## Results

### 2015–2024 changes of pediatric infectious diseases

This retrospective cohort study, conducted at the First Affiliated Hospital of Xiamen University, encompassed pediatric inpatients (≤ 14 years old) admitted from January 2015 to January 2024, excluding cases of COVID-19 infection (Table 1). The majority of patients hailed from southwestern Fujian Province, where the hospital predominantly serves. Out of 57,471 children, there were 23,178 diagnosed cases of infection, with males accounting for 13,840 cases (59.71%).

**Table 1:** Characteristics and distribution, of children with an infectious disease diagnosis.

	2015	2016	2017	2018	2019	2020	2021	2022	2023	2024.1	Total
Total inpatients to pediatricians, n	6,235	6,443	6,986	6,676	7,027	5,569	5,587	5,926	6,534	488	57,471
Children with an infectious disease diagnosis, n (%)	2,615 (41.94)	2,640 (40.97)	2,754 (39.42)	2,706 (40.53)	2,750 (39.13)	1,542 (27.69)	1940 (34.72)	2,848 (48.06)	3,098 (47.41)	285 (58.40)	23,178 (40.33)
Male, n (%)	1,681 (64.28)	1,680 (63.64)	1,752 (63.62)	1,630 (60.24)	1,696 (61.67)	938 (60.83)	1,186 (61.13)	1,326 (46.59)	1,788 (57.71)	163 (57.19)	13,840 (59.71)
Age in months, median (IQR)	22 (7.47–60)	27 (9.43–60)	36 (13–120)	25 (8.97–60)	33 (12–65)	29 (10.63–60)	30 (10.03–57)	34 (10.82–60)	63 (24–84)	71 (40–96)	N/A
0–3 years, n (%)	1,765 (67.50)	1,615 (61.17)	1,391 (50.51)	1,612 (59.57)	1,441 (52.40)	862 (55.90)	1,079 (55.62)	1,195 (41.96)	988 (31.89)	67 (23.51)	12,015 (51.84)
3–6 years, n (%)	445 (17.02)	540 (20.45)	439 (15.94)	664 (24.54)	893 (32.47)	426 (27.63)	583 (30.05)	757 (26.58)	1,119 (36.12)	115 (40.35)	5,981 (25.80)
6–14 years, n (%)	405 (15.49)	485 (18.37)	924 (33.55)	430 (15.89)	416 (15.13)	254 (16.47)	278 (14.33)	896 (31.46)	991 (31.99)	103 (36.14)	5,182 (22.36)

Children can be counted several times as they can have multiple visits with a diagnosis of infectious disease.

Compared to 2015–2019, the number of annual infectious patients reported in 2020–2022 decreased by 583 (21.65%). Particularly, in 2020, the proportion of children diagnosed as community-acquired infections dropped sharply to no more than 30% of all those admitted. Notably, the number of cases crept up with the easing of the NPIs. Besides, a noticeable shift in the age distribution of affected children has been observed after the outbreak of COVID-19. The age group with the highest number of cases transitioned from 0 to 3 years old before the pandemic to 3–6 years old afterward (Table 1).

**Figure 1:**
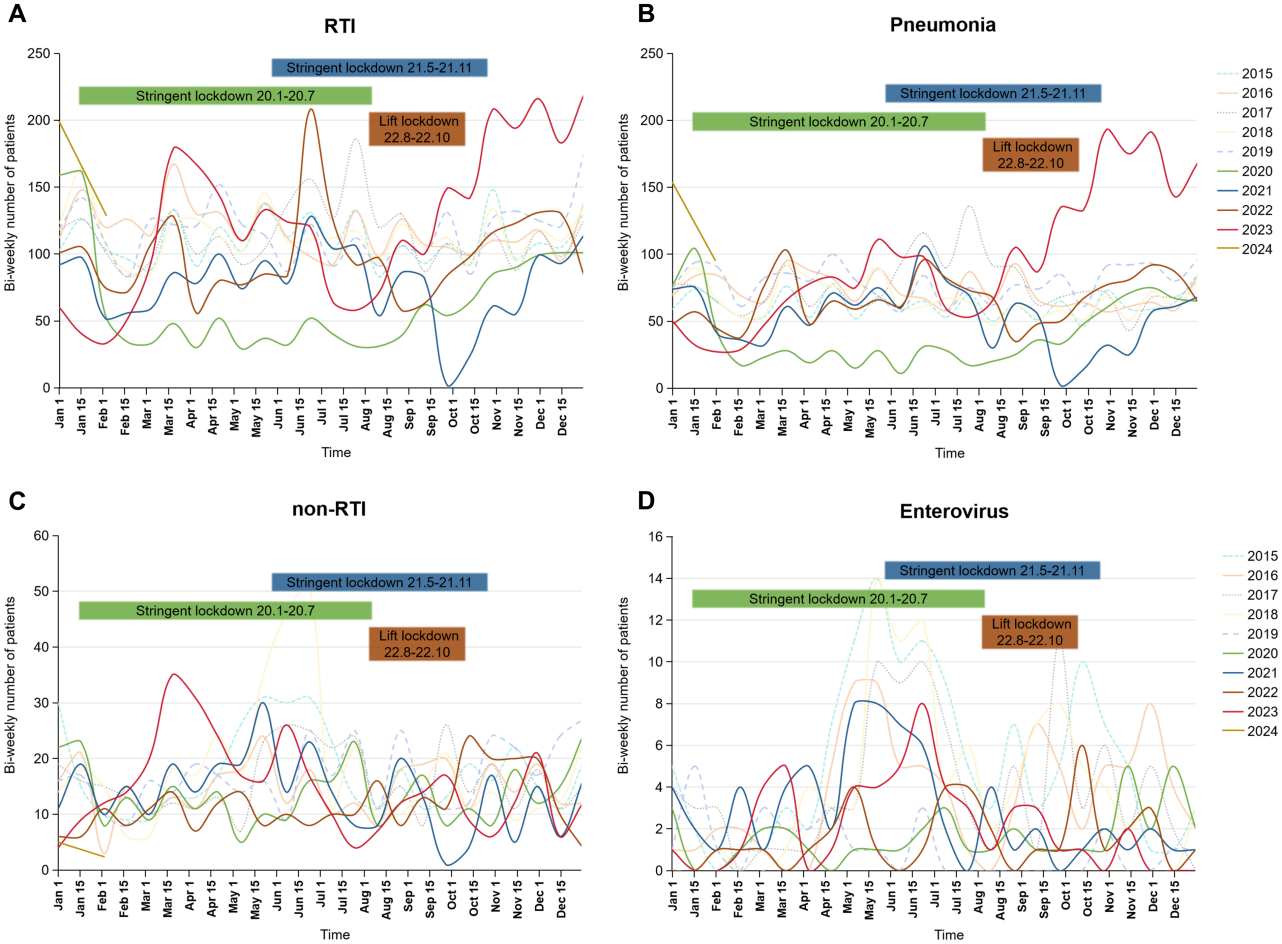
Bi-weekly number of RTI, pneumonia, non-RTI and enteroviral infections during January 2015 to January 2024 in the pediatric inpatient case database. **(A)** RTI, respiratory infection; **(B)** pneumonia; **(C)** non-RTI, non-respiratory tract infection; **(D)** enteroviral infections. The bars labeled “Stringent Lockdown” correspond to the years indicated by their colors: green for 2020 and blue for 2021. The bars labeled “Lift Lockdown” are represented in brown for 2022.

Table 2 presents that in 2020, almost all RTIs monitored, except for non-annual epidemic infections such as influenza-like-illness and bronchitis, showed a downward trend compared to 2015–2019 (OR = 0.62, *p* < 0.0001). As the largest proportion of respiratory infections, the number of pneumonia case declined from 54.29% for 2015 to 2019 to 51.44% immediately after the implementation of NPIs in the end of January 2020 (OR = 0.892, *p* = 0.019), then surged sharply toward the end of 2022 after the cessation of NPIs, with the number of patients markedly exceeding those in previous years, reaching 69.64% in 2023 (OR = 1.913, *p* < 0.0001; Table 2; Figures 1A,B). In addition, the incidence rates have increased for certain non-RTIs such as viral enteritis and urinary tract infections (Table 2, OR from 1.77 to 3.02, *p* < 0.0001). However, overall, there is not a substantial difference in the number of non-respiratory infections before and after the lockdown (Figure 1C). Over the past decade, the seasonal pattern of enterovirus infections has remained consistent, but during the pandemic and post-pandemic period, there has been a reduction in the peak number of cases during the summer months, with the autumn peak appearing later than usual (Figure 1D). Bronchitis and urinary tract infections have shown high incidence rates, with occasional peaks (Supplementary Figure 1), but overall, there is not a substantial seasonal difference during the pandemic and post-pandemic period compared to the years 2015–2019.

**Table 2:** The annual frequencies and percentages of infectious disease from 2020 to January 2024 compared to the 2015–2019 period.

	2015–2019		2020			2021			2022			2023–2024.1		
	*N* = 15,810		*N* = 1,878			*N* = 2,205			*N* = 2,694			*N* = 3,659		
	n (%)	OR	n (%)	OR^b^ [95% CI]	*p* value^a^	n (%)	OR^b^ [95% CI]	*p* value^a^	n (%)	OR^b^ [95% CI]	*p* value^a^	n (%)	OR^b^ [95% CI]	*p* value^a^
RTI, n (%)	13,804 (87.31)	1	1,519 (80.88)	**0.615 [0.543 ~ 0.696]**	**<0.0001**	1875 (85.03)	**0.826 [0.728 ~ 0.937]**	**0.003**	2,379 (89.44)	1.098 [0.967 ~ 1.246]	0.149	3,297 (90.11)	**1.324 [1.176 ~ 1.490]**	**<0.0001**
Tonsillopharyngitis and pharyngitis, n (%)	402 (2.54)	1	28 (1.49)	**0.580 [0.394 ~ 0.853]**	**0.006**	43 (1.95)	0.762 [0.555 ~ 1.047]	0.094	51 (1.92)	**0.740 [0.551 ~ 0.993]**	**0.045**	27 (0.74)	**0.285 [0.193 ~ 0.421]**	**<0.0001**
Pneumonia, n (%)	8,583 (54.29)	1	966 (51.44)	**0.892 [0.810 ~ 0.981]**	**0.019**	1,295 (58.73)	**1.198 [1.095 ~ 1.312]**	**<0.0001**	1,586 (59.62)	**1.205 [1.109 ~ 1.309]**	**<0.0001**	2,548 (69.64)	**1.931 [1.788 ~ 2.086]**	**<0.0001**
Influenza-like illness, n (%)	115 (0.73)	1	57 (3.04)	**4.272 [3.098 ~ 5.890]**	**<0.0001**	18 (0.82)	1.123 [0.682 ~ 1.850]	0.648	165 (6.20)	**8.904 [6.992 ~ 11.340]**	**<0.0001**	347 (9.48)	**14.299 [11.542 ~ 17.714]**	**<0.0001**
Bronchitis, n (%)	1,366 (8.64)	1	114 (6.07)	**0.683 [0.561 ~ 0.832]**	**<0.0001**	135 (6.12)	**0.690 [0.574 ~ 0.828]**	**<0.0001**	188 (7.07)	**0.793 [0.677 ~ 0.929]**	**0.004**	98 (2.68)	**0.291 [0.236 ~ 0.358]**	**<0.0001**
Pertussis, n (%)	44 (0.28)	1	3 (0.16)	0.573 [0.178 ~ 1.848]	0.352	16 (0.73)	**2.619 [1.475 ~ 4.649]**	**0.001**	37 (1.39)	**4.990 [3.216 ~ 7.741]**	**<0.0001**	14 (0.38)	1.376 [0.753 ~ 2.514]	0.299
Bronchiolitis, n (%)	47 (0.30)	1	20 (1.06)	**3.610 [2.135 ~ 6.106]**	**<0.0001**	38 (1.72)	**5.881 [3.826 ~ 9.040]**	**<0.0001**	17 (0.64)	**2.130 [1.221 ~ 3.715]**	**0.008**	48 (1.31)	**4.458 [2.977 ~ 6.676]**	**<0.0001**
Other RTIs, n (%)	3,247 (20.54)	1	331 (17.62)	**0.828 [0.731 ~ 0.938]**	***p* = 0.003**	330 (14.97)	**0.681 [0.602 ~ 0.770]**	**<0.0001**	335 (12.59)	**0.549 [0.487 ~ 0.620]**	**<0.0001**	215 (5.88)	**0.242 [0.209 ~ 0.279]**	**<0.0001**
Non-RTI, n (%)	2006 (12.69)	1	359 (19.12)	**1.626 [1.436 ~ 1.841]**	**<0.0001**	330 (14.97)	**1.211 [1.068 ~ 1.374]**	**0.003**	315 (11.84)	0.911 [0.803 ~ 1.034]	0.149	362 (9.89)	**0.756 [0.671 ~ 0.850]**	**<0.0001**
Otitis, n (%)	104 (0.66)	1	11 (0.59)	0.890 [0.477 ~ 1.659]	0.713	17 (0.77)	1.173 [0.701 ~ 1.963]	0.543	19 (0.71)	1.073 [0.657 ~ 1.752]	0.779	20 (0.55)	0.830 [0.514 ~ 1.341]	0.447
Gastroenteritis, n (%)	265 (1.68)	1	55 (2.93)	**1.770 [1.318 ~ 2.376]**	**<0.0001**	51 (2.31)	**1.389 [1.026 ~ 1.881]**	**0.034**	46 (1.73)	1.019 [0.743 ~ 1.397]	0.907	48 (1.31)	0.780 [0.572 ~ 1.063]	0.115
Enteroviral infections, n (%)	429 (2.71)	1	39 (2.08)	0.760 [0.546 ~ 1.059]	0.105	69 (3.13)	1.158 [0.895 ~ 1.499]	0.265	37 (1.39)	**0.499 [0.356 ~ 0.700]**	**<0.0001**	51 (1.39)	**0.507 [0.378 ~ 0.679]**	**<0.0001**
Chickenpox, n (%)	15 (0.09)	1	1 (0.05)	0.561 [0.074 ~ 4.249]	0.576	4 (0.18)	1.914 [0.635 ~ 5.771]	0.249	3 (0.11)	1.174 [0.340 ~ 4.058]	0.8	1 (0.03)	0.288 [0.038 ~ 2.180]	0.228
Urinary tract infection, n (%)	555 (3.51)	1	186 (9.90)	**3.022 [2.540 ~ 3.594]**	**<0.0001**	127 (5.76)	**1.680 [1.378 ~ 2.048]**	**<0.0001**	159 (5.98)	**1.724 [1.438 ~ 2.067]**	**<0.0001**	135 (3.69)	1.053 [0.869 ~ 1.275]	0.598
Viral encephalitis, n (%)	616 (3.90)	1	63 (3.35)	0.856 [0.658 ~ 1.115]	0.249	49 (2.22)	**0.561 [0.418 ~ 0.752]**	**<0.0001**	50 (1.88)	**0.466 [0.349 ~ 0.624]**	**<0.0001**	99 (2.71)	**0.686 [0.553 ~ 0.851]**	**0.001**
Other, n (%)	22 (0.14)	1	4 (0.21)	1.532 [0.527 ~ 4.450]	0.433	13 (0.59)	**4.256 [2.141 ~ 8.461]**	**<0.0001**	1 (0.04)	0.266 [0.036 ~ 1.978]	0.196	8 (0.22)	1.572 [0.700 ~ 3.535]	0.273

COVID-19 and other infectious disease diagnoses are not included in the Table. 95% CI, 95% confidence interval; RTI, respiratory tract infection; non-RTI, non-respiratory tract infection.

a Significant *p* values (<0·05) are shown in bold.

b For each disease, the dependent variable is dichotomic: disease/no disease. The OR calculations are based on the comparison of each year (2020–2024.1) to the reference (2015–2019).

Our research findings indicate that the frequency and proportion of respiratory infections among hospitalized children has been weakened by the implementation of NPIs. Following the withdrawal, there was a significant rebound in the number and proportion of respiratory infections, with pneumonia as the representative condition, compared to pre-pandemic levels. Consequently, we conducted further analysis of respiratory infections.

### Trends of respiratory pathogens

In 2019, the hospital saw 1944 patients with respiratory pathogen infections. During the 2020 pandemic, only 934 cases were reported, rising to 2031 post-NPI lifting in 2023. Compared to 2019, significant epidemiological variations in the prevalence of common respiratory pathogens were observed during and after the COVID-19 pandemic. Notably, MP, a major pathogen, constituted about one-third of cases yearly, peaking at 35.79% in 2019 (Supplementary Table 2). While bacterial pathogens had low and stable detection rates, SP and HI saw notable outbreaks post-pandemic (*p* < 0.0001). Viral pathogen fluctuation was observed, with a decrease during the pandemic and a recovery afterward, with RSV cases surging post-pandemic.

**Figure 2:**
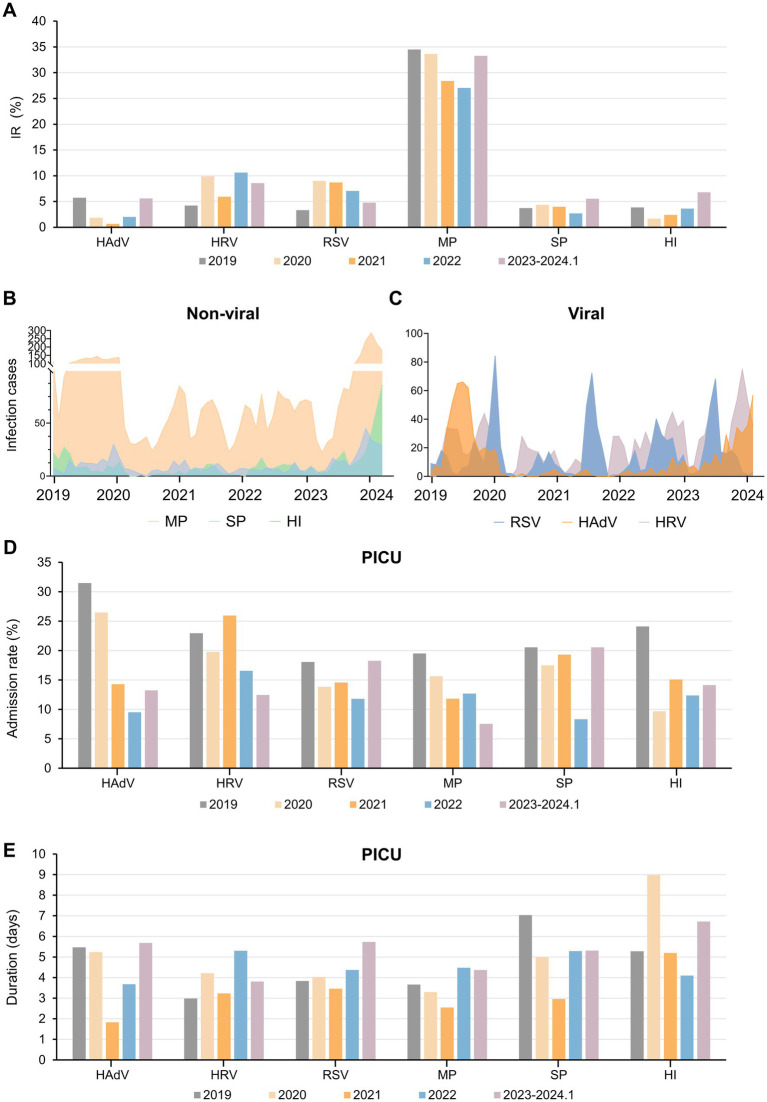
Overview of the IR, infection cases and PICU admissions of six respiratory pathogens during the past 5 years. **(A)** IR of six important respiratory pathogens pre-, during and post-epidemic. **(B,C)** Infection cases of several non-viral **(B)** and viral **(C)** respiratory pathogens before and after the epidemic. Admission rate to the PICU **(D)** and duration of PICU **(E)** for each type of pathogen-infected patients. Abbreviations: IR, infection rate; PICU, Pediatric Intensive Care Unit; HAdV, human adenovirus; HRV, human rhinoviruses; RSV, respiratory syncytial virus; MP, *Mycoplasma pneumoniae*; SP, *Streptococcus pneumoniae*; HI, *Haemophilus influenzae*.

Six pathogens were selected with the highest number of IRs. During the pandemic, NPIs reduced the IR of these pathogens, rebounding post-NPI lifting, surpassing pre-pandemic levels. MP had the highest IR at the average of 31.38%, with SP and HI seeing significant increases post-NPI measures in 2023, reaching 138 and 246% of the annual averages, respectively (Figure 2A). The prevalence of MP infections exhibited multiple peaks annually, with stable monthly cases around 140 in the latter half of 2019 (Figure 2B). In 2023, there was a significant surge in infections, reaching as high as 285 cases in November. Similar seasonal patterns were observed for SP, HI, and HAdV (Figures 2B,C), characterized by low IR before and during the initial stages of the pandemic, followed by a rapid increase post-lifting of NPIs. RSV transmission displayed clear winter peaks, persisting until the following March–April period. Notably, the peak of RSV infections in 2020 significantly declined, but following the peaks in 2023 advanced to the summer months, while HRV transmission persisted year-round, with prolonged period during the later stages of the pandemic (Figure 2C).

Consistent with the significant reduction in the number and IR of various pathogens, the pandemic led to reduced PICU admission rates among infected children (Figure 2D). From 2019 to 2024, the duration of PICU duration for pediatric patients exhibited an overall decreasing trend, followed by an increase, except for HI (Figure 2E). Despite MP’s highest IR, PICU admission rates were initially low, with short total hospital stay compares to other pathogens (Figure 2D; Supplementary Figure 2). However, post-pandemic, the duration of PICU stay for MP patients increased, indicating the occurrence of more severe respiratory conditions (Figure 2E).

### Age profiles of *Mycoplasma pneumoniae* infection

**Figure 3:**
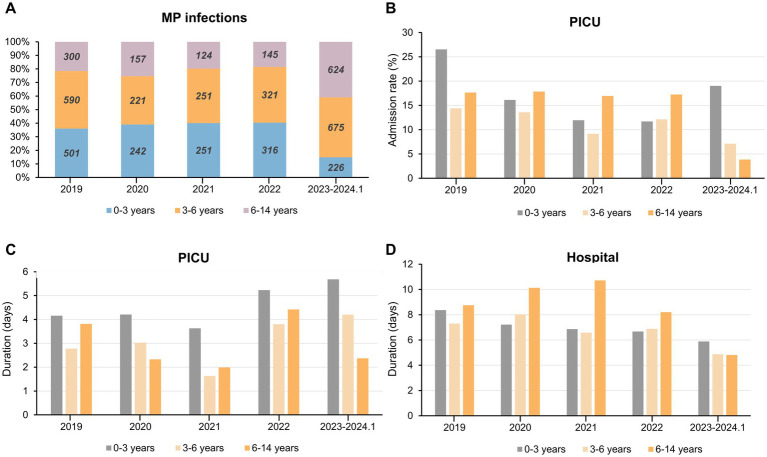
Overview of MP infection and PICU admission among children in different age groups during the past 5 years. **(A)** Yearly proportion and cases of children infected with MP in different age groups. **(B–D)** The admission rate to PICU **(B)** duration of PICU **(C)** and duration of total hospital stay **(D)** for children of different age groups of MP infection. Abbreviations: MP, *Mycoplasma pneumoniae*; PICU, Pediatric Intensive Care Unit.

Our study revealed the age distribution of MP infections among children (Figure 3). The age distribution of MP infections remained stable till the withdrawal of NPIs. Preschool children (aged 1 month to 6 years) were at higher risk of respiratory diseases caused by MP. Following NPIs relaxation, there was a notable decrease in infections among infants aged 1 month to 3 years (from 35 to 15%), accompanied by a significant increase among children aged 6 to 14 years (from 20 to 40%; Figure 3A).

Infants aged 1 month to 3 years exhibited the highest PICU admission rate and longest PICU duration both pre- and post-pandemic compared to other age groups (Figures 3B,C). For children aged 3 to 6 years, these two metrics along with total duration of hospitalization gradually declined during NPIs. Interestingly, for patients aged 6 years and older, there was a swift decline in these three metrics following the cessation of NPIs in 2023 (Figures 3B–D). In children afflicted with respiratory illnesses stemming from MP infection, the PICU duration showed a nadir in 2021 and peaked in 2023. Among children aged 6 and below, there was an increment of approximately 1.3 days compared to 2019 and 2.1 days compared to 2021 (Figure 3C). During the period of NPIs, the average duration of hospitalization for children aged 6–14 years increased by approximately 2 day and experienced a rapid decline in 2023 (Figure 3D). In summary, after the pandemic, children under 3 experienced higher severity of MP infections following the withdrawal of NPIs, evident in longer PICU duration as well as MP infections being more prevalent among older children.

### Changes of coinfection pattern with *Mycoplasma pneumoniae*

**Figure 4:**
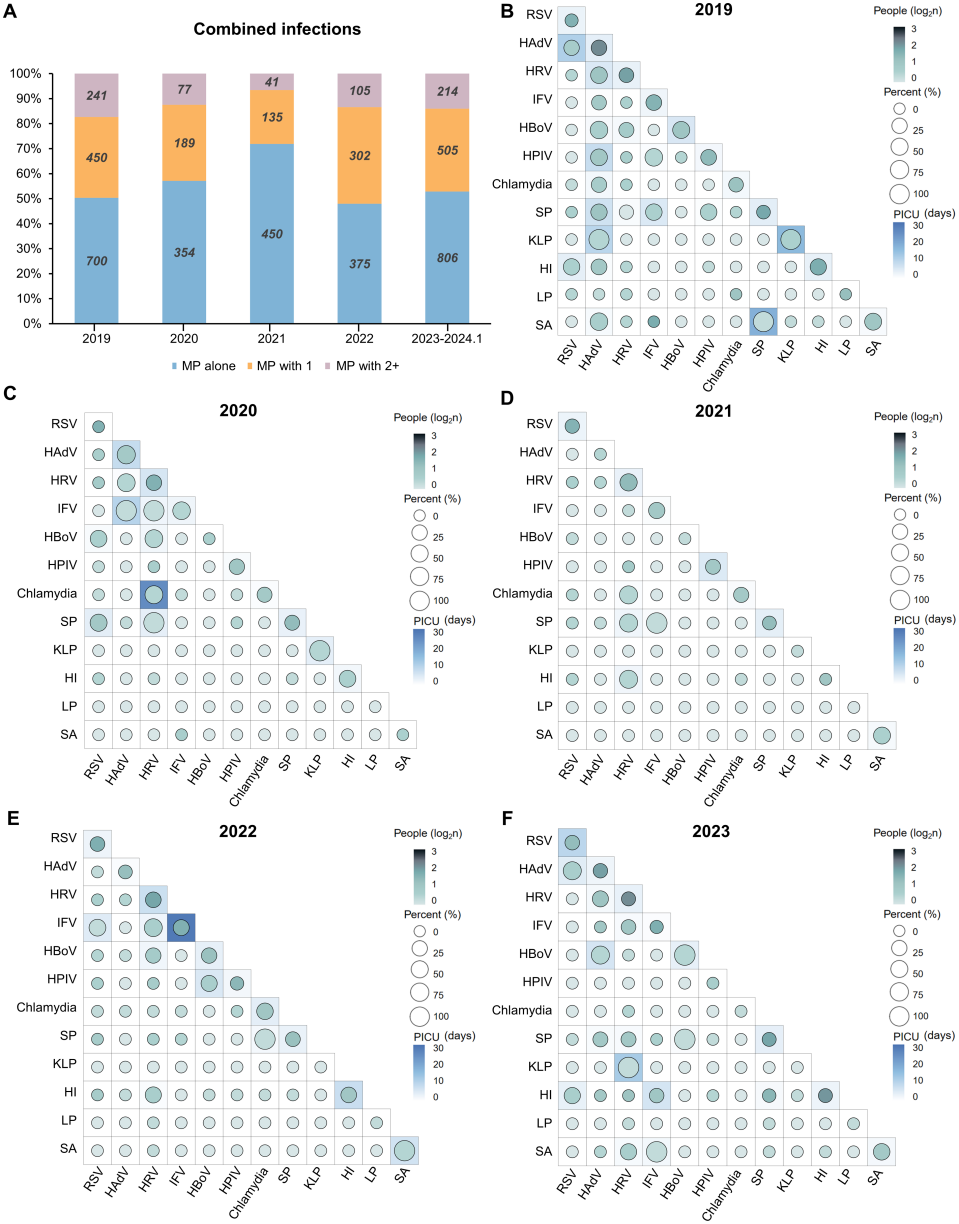
Landscape of mixed infections involving MP. **(A)** The proportion and cases of combined infections involving MP and multiple pathogens. **(B–F)** The association between the types of mixed infections involving MP and the PICU admission rate and duration of PICU from 2019 to January 2024 (Data from 2023 and January 2024 were consolidated). Each square represents the presence of additional pathogens in patients infected with MP; for example, “RSV alone (top left)” indicates mixed infection with MP and RSV. The circle color denotes the count of individuals coinfected with MP, with the number undergoing a log_2_ transformation to enhance differentiation. The size of the circles and the background color of the squares indicates the PICU admission rate (%) and the PICU duration (days) resulting from coinfections of one or both pathogens with MP, respectively. Abbreviations: MP, *Mycoplasma pneumoniae*; PICU, Pediatric Intensive Care Unit; RSV, respiratory syncytial virus; HAdV, human adenovirus; HRV, human rhinoviruses; IFV, influenza virus; HBoV, human bocavirus; SP, *Streptococcus pneumoniae*; KLP, *Klebsiella pneumoniae*; HI, *Haemophilus influenzae*; LP, *Legionella pneumophila*; SA, *Staphylococcus aureus*.

Significant rates of coinfection involving MP with other bacteria and viruses have been reported, leading to severe illnesses (17). Therefore, we conducted an investigation into cases of coinfection involving MP and other pathogens (Figure 4). From January 2019 to January 2024, the number and proportion of mixed infections involving MP showed a decreasing trend followed by an increase. In 2021, there were only 176 cases of mixed infections, accounting for 28.11% of all cases. However, from 2023 to January 2024, the number of mixed infection cases peaked at 719, accounting for 43.6%, resembling the levels observed in 2019 (Figure 4A). In 2019 and 2023, the predominant coinfecting pathogens with MP were HRV and HAdV. Conversely, during the pandemic, RSV coinfection was most prevalent. Notably, in 2019, HRV and SP were the most common coinfecting agents; in 2020 and 2021, RSV and SP prevailed; in 2022, RSV and IFV were prominent; and in 2023, IFV and HAdV were predominant (Figures 4B–F).

Before the pandemic, more severe cases of mixed infections (longer PICU duration) often occurred due to coinfection of MP alongside one or two other bacterial pathogens, such as SP or KLP, resulting in a PICU hospital stay of up to 17.65 days, associated with a PICU admission rate exceeding 50% (Figure 4B). The year 2022 was particularly notable, as the outbreak of influenza led to cases of IFV coinfection, which were more severe compared to previous years, with some cases even requiring a PICU stay of up to 24.04 days (totaling 52 cases) (Figure 4E). Aligned with the notable surge in the IRs of SP and HI, in 2023 and January 2024 (Figure 2), the count of patients coinfected with MP & HI surpassed 100. Likewise, the number of patients with concurrent infections of MP & SP & HI reached 30. However, their PICU hospital stays ranged merely from 1 to 3 days (Figure 4F). The analysis suggests that the implementation of NPIs has not only effectively reduced the number of cases of MP coinfections, but also alleviated their severity, despite the occurrence of special pathogen outbreaks each year. Bacterial coinfections with MP, compared to viral pathogens, were markedly affected by NPIs during 5 years.

## Discussion

Our study stands as the sole investigation focusing on the trends of pediatric infectious diseases in the southeastern part of Fujian Province over a span of 10 years (2015–2024). This research not only delved into the impact of NPIs on the transmission patterns of 13 common respiratory pathogens but also placed emphasis on MP. We analyzed its age distribution characteristics as well as both dual and triple coinfections revolving around MP.

China’s implementation of NPIs resulted in a significant decrease in pediatric RTIs, including pneumonia, bronchitis, and tonsillitis, in Xiamen City since January 2020. However, the lifting of these measures led to a rapid surge in RTIs, particularly pneumonia. Conversely, the incidence of non-RTIs remained relatively stable throughout the pandemic, underscoring the effectiveness of NPI policies in mitigating respiratory diseases transmitted via droplets and reducing disease burden (18–20). Besides severe restrictions on public movement and limited access to hospitals, NPIs, such as mask-wearing, home isolation, and enhanced surface cleaning and disinfection were instrumental in curbing disease transmission by interrupting transmission chains and reducing the pathogen load in the environment, thereby achieving effective disease control.

Over the 10-year period, with technological advancements, hospitals progressively optimized pathogen detection methods. Our observation of the overall trends in respiratory pathogens revealed that despite the presence of similar seasonal distribution patterns before and after the COVID-19 pandemic, the IR of common bacteria and most viruses saw a significant increase post-pandemic, surpassing pre-pandemic levels. Of note, although RSV admission rate to the PICU decreased during the pandemic period, the IR increased. This could be attributed to the relatively reduced IR of other pathogens or suggest that RSV derived limited benefit from NPIs. Conclusions from researches on the epidemiological characteristics of RSV before and after the COVID-19 pandemic differ between China and other countries. While most foreign studies (21–23) indicate that NPI implementation effectively curbs RSV transmission, the majority of Chinese researchers (24–26) believe RSV continues to be highly transmissible during the pandemic. This discrepancy may be attributed to the prolonged survival of RSV in hosts and its strong capacity for rapid transmission, or alterations in RSV spectrum due to preventive measures against COVID-19. In addition, due to the special climate of Xiamen, RSV outbreaks were concentrated in summer, which was inconsistent with the period when the NPIs were most strictly implemented in 2020, this may also explain the differences in the transmission.

MP, as a prokaryotic organism without cell walls and unable to self-replicate, is one of the clinically significant smallest pathogens in children and a major causative agent of community-acquired pneumonia (27, 28). Our work indicates that MP had the highest IR both before and after the pandemic, with an average of 43.08%. Although its overall numbers of infection decreased significantly during the pandemic, along with reduced PICU admission rates and durations, it remains the most prevalent of all respiratory tract pathogens. Moreover, it re-emerged and led to another outbreak after the pandemic. And as a result, the PICU duration soared, indicating a more severe respiratory diseases. These findings are consistent with previous studies (29–32), reaffirming the effectiveness of NPI implementation in preventing respiratory tract infections. Additionally, we observed a higher IR among preschool children in previous studies, whereas after the end of the pandemic, IR increased among school-aged children, possibly due to susceptible individuals being protected from infection by NPIs, leading to a higher likelihood of infection after their cessation, resulting in an overall increase in the average age of patients.

However, it is worth noting that although there was a resurgence of MP infections following the end of the pandemic, the study results showed a decrease in both the PICU admission rate and the average length of hospital stay for children aged 3 and above. This could be attributed to the severity of MP infections in 2023, which were characterized by extensive pulmonary involvement, resulting in a higher number of hospital admissions being required. Due to limited pediatric ward capacity, efforts were made to promptly treat more patients by increasing hospital bed capacity, providing early treatment guidance to all patients during outpatient visits, aiming to shorten the length of hospital stay, increase bed turnover rate, and facilitate early discharge with outpatient follow-up visits. In contrast, children under 3 years old often exhibit severe symptoms such as dyspnea, respiratory distress, and septicemia, necessitating admission to the PICU for advanced respiratory support and treatment.

Furthermore, we found a high likelihood of MP mixed infections, primarily with viruses, which significantly decreased in 2021, followed by a peak in cases in 2023. Studies (17) suggest that apart from MP itself being one of the most important potential coinfecting pathogens, the surge in macrolide resistance contributes to increased susceptibility to mixed infections. Evidence indicates that macrolide resistance in Asian countries, especially China and Japan, can exceed 90%, primarily due to inappropriate or excessive antibiotic use (33–35). Additionally, mixed infections increase the incidence of severe MP pneumonia, leading to prolonged duration of PICU. Despite the rise in MP mixed bacterial infections post-2023, PICU admission and hospital stay decreased, possibly due to enhanced bed turnover rates and increased utilization of tetracyclines and fluoroquinolones to counteract rising macrolide resistance, thereby partially alleviating the severity of bacterial infections.

Our study also revealed that post-pandemic, the age of patients suffering from respiratory infections increased, with the age group most affected shifting from 0 to 3 years before the COVID-19 pandemic to 3–6 years after the pandemic. Delayed immunity may be one reason for this transition, as the immune system of children, after being “compensating for lost time” upon exposure to certain pathogens post-NPIs, could lead to the resurgence of infectious diseases (36). Furthermore, implementation of NPIs has led to prolonged low exposure to specific pathogens in young children during the COVID-19 pandemic, resulting in a higher susceptibility to infections, leading to an “immunity debt” and subsequent outbreaks of infectious diseases in many countries post-COVID-19, along with an increase in the median age of onset (37–40). However, the mechanism of immunity debt remains controversial, with some scholars suggesting that the outbreak of specific pathogens may be related to the comprehensive and long-term damage to the immune system after SARS-CoV-2 infection, leading to a decrease in the population’s immune barrier (36). Further in-depth research is warranted on the mechanism of immunity debt.

Several limitations exist in our study. Firstly, being a single-center study, its findings may not be generalizable to other regions despite the large sample size. Secondly, focusing solely on hospitalized patients limits the understanding of the overall situation among children in the region. Many children with infections who do not require hospitalization are not represented in our data. Therefore, our findings may not fully capture the spectrum of disease severity or the true incidence and prevalence of infections in the broader community. Lastly, while our results provide population-level insights, they may not apply to individual cases due to differences in host factors (such as age, immune status, or genetic predispositions) and environmental influences. The interaction between these factors may lead to variability in how different individuals respond to the same pathogen or combination of pathogens.

In conclusion, our study affirms the effectiveness of NPIs in preventing respiratory infections. However, further mechanistic research is needed on delayed immunity and immunity debt post-pandemic. MP, as a special pathogen, continues to exhibit cyclical outbreaks globally, with its increasing resistance and the risks associated with resistance warranting attention.

## Brief summary

Most common infectious diseases and respiratory pathogens declined under NPIs but rebounded notably in 2023. The MP outbreak was characterized by a higher severity of illness in children under 3 years of age and a rise in the number of cases among older children. NPIs reduced MP coinfections primarily by decreasing the MP coinfections with other bacteria.

## Data Availability

The original contributions presented in the study are included in the article/Supplementary material, further inquiries can be directed to the corresponding authors.
