# Supplementary material for: Respiratory pathogen analysis in pediatric inpatients unraveled the infection pattern of Mycoplasma pneumoniae post the COVID-19 pandemic
Source: Front Public Health. 2024 Oct 9;12:1437508. doi: 10.3389/fpubh.2024.1437508 (PMC11496064; doi:10.3389/fpubh.2024.1437508)
Supplement: Supplementary file 1 [file Data_Sheet_1.docx]

**Supplement:**

**Respiratory pathogen analysis in pediatric inpatients unraveled the infection pattern of *mycoplasma pneumoniae* post the COVID-19 pandemic**

| **Supplementary Table 1.** International Classification of Diseases (ICD) 10th edition used for the study | Page 2 |
| --- | --- |
| **Supplementary Table 2.** Frequencies and percentages of respiratory pathogens in hospitalized children of RTI from 2019 to January 2024 | Page 3 |
| **Supplementary Figure 1.** Bi-weekly number of bronchitis and urinary tract infection during January 2015 to January 2024 in the pediatric inpatient case database | Page 6 |
| **Supplementary Figure 2.** Duration of hospitalization for children infected with different pathogens over the past 5 years | Page 7 |

**Supplementary Table 1.** International Classification of Diseases (ICD) 10th edition used for the study.

|  | **Diagnosis** | **ICD codes** |
| --- | --- | --- |
| **RTI** | Tonsillopharyngitis and pharyngitis | J02 J02.0 J02.9 J03 J03.9 |
|  | Pneumonia | J12.9 J15 J15.9 J18 |
|  | Influenza-like illness | J10 J10.1 J11 |
|  | Bronchitis | J20 |
|  | Pertussis | A37 |
|  | Bronchiolitis | J21 |
|  | Other RTIs | J06 |
| **non-RTI** | Otitis | H65 H65.1 H65.4 H65.49 H66 H66.0 H66.4 H73.0 H92.1 |
|  | Gastroenteritis | A08.4 A08.2 A08.5 A08.0 A09 |
|  | Enteroviral infections | R21 B08.4 B08.5 B97.1 |
|  | Chickenpox | B01 B02 |
|  | Urinary tract infection | N10 N30 N30.0 N39.0 |
|  | Viral encephalitis | A86 |
| **Others** | | A38 A38.9 L53.8 |

**Legend:** The table shows the International Classification of Diseases (ICD) 10th edition used for the study.

**Abbreviations:** Respiratory tract infection (RTI), non-Respiratory tract infection (non-RTI), International Classification of Diseases (ICD).

**Supplementary Table 2. Frequencies and percentages of respiratory pathogens in hospitalized children of RTI from 2019 to January 2024.**

| **Respiratory pathogens** | **Abbreviation** | **2019** | | **2020** | | | **2021** | | | **2022** | | | | **2023-2024.1** | | |
| --- | --- | --- | --- | --- | --- | --- | --- | --- | --- | --- | --- | --- | --- | --- | --- | --- |
|  |  | **N=3887** | | **N=1823** | | | **N=2205** | | | **N=2774** | | | | **N=4583** | | |
|  |  | **n(%)** | **OR** | **n(%)** | **OR^b^**  **[95% CI]** | ***P* value^a^** | **n(%)** | **OR^b^**  **[95% CI]** | ***P* value^a^** | | **n(%)** | **OR^b^**  **[95% CI]** | ***P* value^a^** | **n(%)** | **OR^b^**  **[95% CI]** | ***P* value^a^** |
| *Mycoplasma pneumoniae* | MP | 1391  (35.79) | 1 | 620  (34.01) | 0.925 [0.823~1.040] | 0.19 | 626  (28.39) | **0.711**  **[0.635 ~ 0.797]** | **<0.0001** | 782  (28.19) | | **0.704**  **[0.634 ~ 0.783]** | **<0.0001** | 1525  (33.28) | **0.895**  **[0.818~ 0.979]** | **0.015** |
| Chlamydia | Chlamydia | 76  (1.96) | 1 | 40  (2.19) | 1.125  [0.764 ~ 1.657] | 0.551 | 51  (2.31) | 1.187  [0.829 ~ 1.700] | 0.348 | 57  (2.05) | | 1.052  [0.744 ~ 1.488] | 0.775 | 26  (0.57) | **0.286**  **[0.183 ~ 0.448]** | **<0.0001** |
| **Viral pathogens** | | 1272  (29.90) | 1 | 551  (30.23) | 0.891  [0.790 ~ 1.004] | 0.059 | 452  (20.50) | **0.53**  **[0.469 ~ 0.600]** | **<0.0001** | 1123(40.48) | | **1.398**  **[1.264 ~ 1.547]** | **<0.0001** | 1358  (29.63) | **0.866**  **[0.789 ~ 0.949]** | **0.002** |
| Influenza virus | IFV | 100  (1.29) | 1 | 46  (2.52) | 0.980  [0.688 ~ 1.396] | 0.912 | 16  (0.73) | **0.277**  **[0.163 ~ 0.470]** | **<0.0001** | 139  (5.01) | | **1.998**  **[1.538 ~ 2.595]** | **<0.0001** | 251  (5.48) | **2.194**  **[1.733 ~ 2.778]** | **<0.0001** |
| Human adenovirus | HAdV | 374  (9.62) | 1 | 34  (1.87) | **0.179**  **[0.125 ~ 0.255]** | **<0.0001** | 14  (0.63) | **0.06**  **[0.035 ~ 0.103]** | **<0.0001** | 58  (2.09) | | **0.201**  **[0.151 ~ 0.266]** | **<0.0001** | 257  (5.61) | **0.558**  **[0.473 ~ 0.658]** | **<0.0001** |
| Human rhinoviruses | HRV | 278  (7.15) | 1 | 182  (9.98) | **1.440[1.184 ~ 1.751]** | **<0.0001** | 131  (5.94) | 0.82  [0.662 ~ 1.016] | 0.07 | 307  (11.07) | | **1.616**  **[1.363 ~ 1.915]** | **<0.0001** | 393  (8.58) | **1.218**  **[1.038 ~ 1.429]** | **0.016** |
| Human metapneumovirus | HMPV | 43  (1.11) | 1 | 21  (1.15) | 1.042[0.616 ~ 1.761] | 0.878 | 10  (0.45) | **0.407**  **[0.204 ~ 0.812]** | **0.011** | 136  (4.90) | | **4.609**  **[3.259 ~ 6.517]** | **<0.0001** | 18  (0.39) | **0.352**  **[0.203 ~ 0.612]** | **<0.0001** |
| Respiratory syncytial virus | RSV | 154  (3.96) | 1 | 166  (9.11) | **2.428[1.936 ~ 3.047]** | **<0.0001** | 192  (8.71) | **2.312**  **[1.858 ~ 2.878]** | **<0.0001** | 204  (7.35) | | **1.924**  **[1.552 ~ 2.386]** | **<0.0001** | 219  (4.78) | **1.216**  **[0.985 ~ 1.502]** | **0.068** |
| Human bocavirus | HBoV | 67  (1.72) | 1 | 26  (1.43) | 0.825[0.523 ~ 1.302] | 0.408 | 13  (0.59) | **0.338**  **[0.186 ~ 0.614]** | **<0.0001** | 76  (2.74) | | **1.606**  **[1.152 ~ 2.239]** | **0.005** | 12  (0.26) | **0.150**  **[0.081 ~ 0.277]** | **<0.0001** |
| Human parainfluenza virus | HPIV | 137  (3.52) | 1 | 65  (3.57) | 1.012  [0.749 ~ 1.367] | 0.938 | 45  (2.04) | **0.570**  **[0.406 ~ 0.802]** | **0.001** | 155  (5.59) | | **1.62**  **[1.280 ~ 2.050]** | **<0.0001** | 33  (0.72) | **0.199**  **[0.135 ~ 0.291]** | **<0.0001** |
| Other viruses |  | 119  (1.53) | 1 | 11  (0.60) | **0.192**  **[0.103 ~ 0.357]** | **<0.0001** | 31  (1.40) | **0.452**  **[0.303 ~ 0.673]** | **<0.0001** | 48  (1.73) | | **0.558**  **[0.397 ~ 0.782]** | **0.001** | 175  (3.82) | 1.257  [0.992 ~ 1.593] | 0.058 |
| **Bacterial pathogens** | | 485  (12.48) | 1 | 190  (10.42) | **0.816**  **[0.683 ~ 0.975]** | **0.025** | 223  (10.11) | **0.789**  **[0.667 ~ 0.934]** | **0.006** | 313  (11.29) | | 0.892  [0.767 ~ 1.038] | 0.139 | 780  (17.02) | **1.439**  **[1.273 ~ 1.626]** | **<0.0001** |
| *Streptococcus pneumoniae* | SP | 144  (3.70) | 1 | 80  (4.39) | 1.193  [0.902 ~ 1.577] | 0.215 | 88  (3.99) | 1.080  [0.824 ~ 1.416] | 0.575 | 78  (2.81) | | **0.752**  **[0.568 ~ 0.995]** | **0.046** | 253  (5.52) | **1.519**  **[1.232 ~ 1.872]** | **<0.0001** |
| *Haemophilus influenzae* | HI | 139  (3.58) | 1 | 31  (1.70) | **0.466**  **[0.315 ~ 0.691]** | **<0.0001** | 53  (2.40) | **0.664**  **[0.482 ~ 0.915]** | **0.012** | 105  (3.79) | | 1.061  [0.819 ~ 1.373] | 0.654 | 311  (6.79) | **1.963**  **[1.600 ~ 2.409]** | **<0.0001** |
| *Staphylococcus aureus* | SA | 32  (0.82) | 1 | 13  (0.71) | 0.865  [0.453 ~ 1.653] | 0.661 | 10  (0.45) | 0.549  [0.269 ~ 1.119] | 0.099 | 26  (0.94) | | 1.14  [0.678 ~ 1.917] | 0.622 | 56  (1.22) | 1.49  [0.963 ~ 2.306] | 0.073 |
| *Pseudomonas aeruginosa* | PA | 15  (0.39) | 1 | 7  (0.38) | 0.995  [0.405 ~ 2.445] | 0.991 | 7  (0.32) | 0.822  [0.335 ~ 2.019] | 0.669 | 10  (0.36) | | 0.934  [0.419 ~ 2.082] | 0.867 | 19  (0.41) | 1.075  [0.545 ~ 2.118] | 0.835 |
| Other bacteria |  | 155  (3.99) | 1 | 59  (3.24) | 0.805  [0.594 ~ 1.093] | 0.164 | 65  (2.95) | **0.731**  **[0.545 ~ 0.982]** | **0.037** | 94  (3.39) | | 0.845  [0.651 ~ 1.096] | 0.204 | 141  (3.08) | **0.764**  **[0.606 ~ 0.964]** | **0.023** |
| **Unknown** | | 663  (17.06) | 1 | 422  (23.15) | **1.465**  **[1.277 ~ 1.680]** | **<0.0001** | 853  (38.68) | **3.068**  **[2.722 ~ 3.458]** | **<0.0001** | 499  (17.99) | | 1.067  [0.938 ~ 1.212] | 0.323 | 894  (19.51) | **1.178**  **[1.055 ~ 1.317]** | **0.004** |

**Legend:** Children can be counted several times as they can have multiple pathogens of RTI.

^a^ Significant P value (<0.05) are shown in bold. Pathogens of COVID-19 and other infectious diseases are not included in the Table.

^b^ For each pathogen, the dependent variable is dichotomic: positive/negative. The OR calculations are based on the comparison of each year (2020 to 2024.1) to the reference (2019).

**Abbreviations:** Confidence interval (95% CI), Odds ratio (OR), Respiratory tract infection (RTI), non-Respiratory tract infection (non-RTI).

**Supplementary Figure 1.** Bi-weekly number of bronchitis and urinary tract infection during January 2015 to January 2024 in the pediatric inpatient case database.


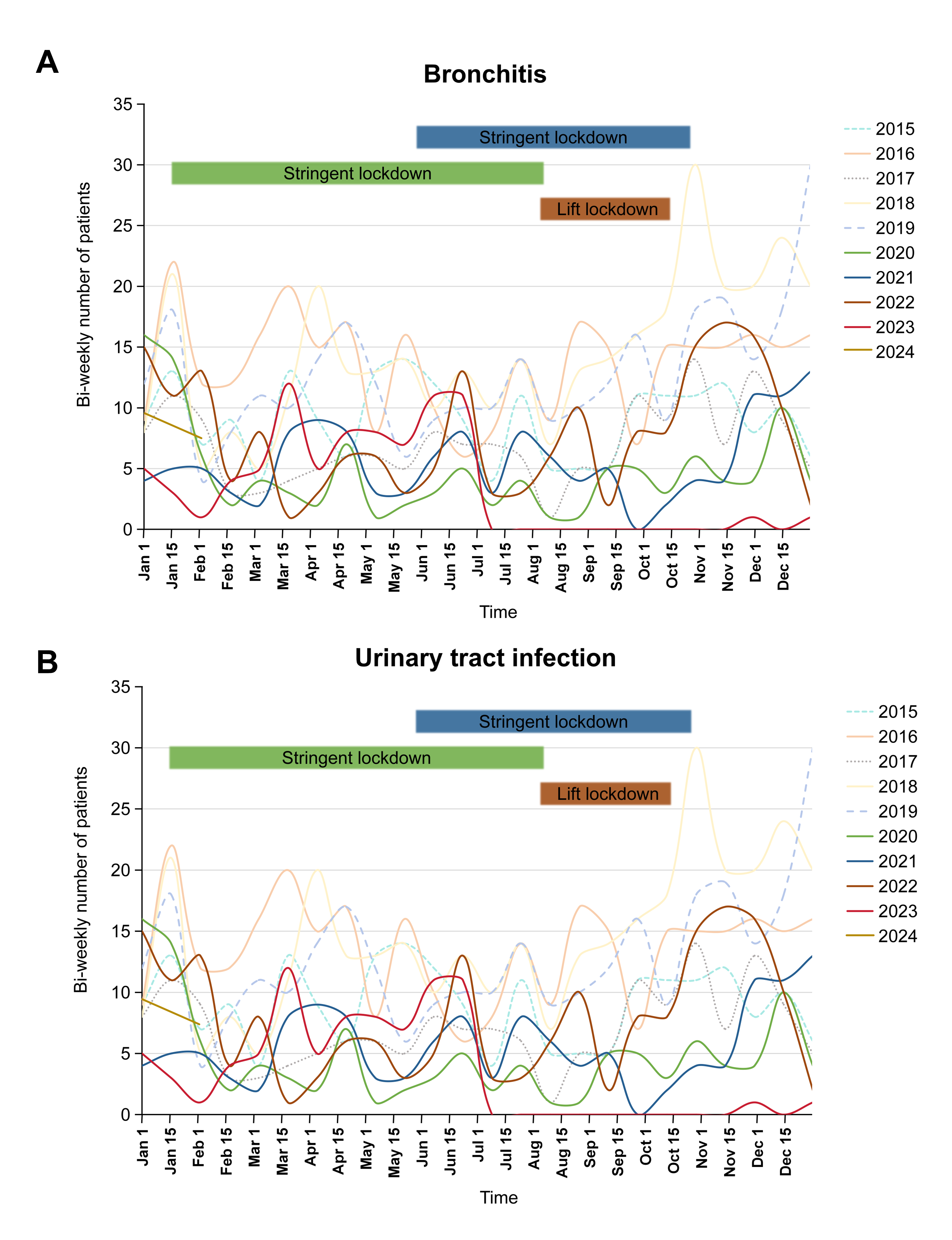


**Legend:** Bi-weekly number of bronchitis and urinary tract infection during January 2015 to January 2024 in the pediatric inpatient case database. A, bronchitis; B, urinary tract infection.

**Supplementary Figure 2.** Duration of hospitalization for children infected with different pathogens over the past 5 years.


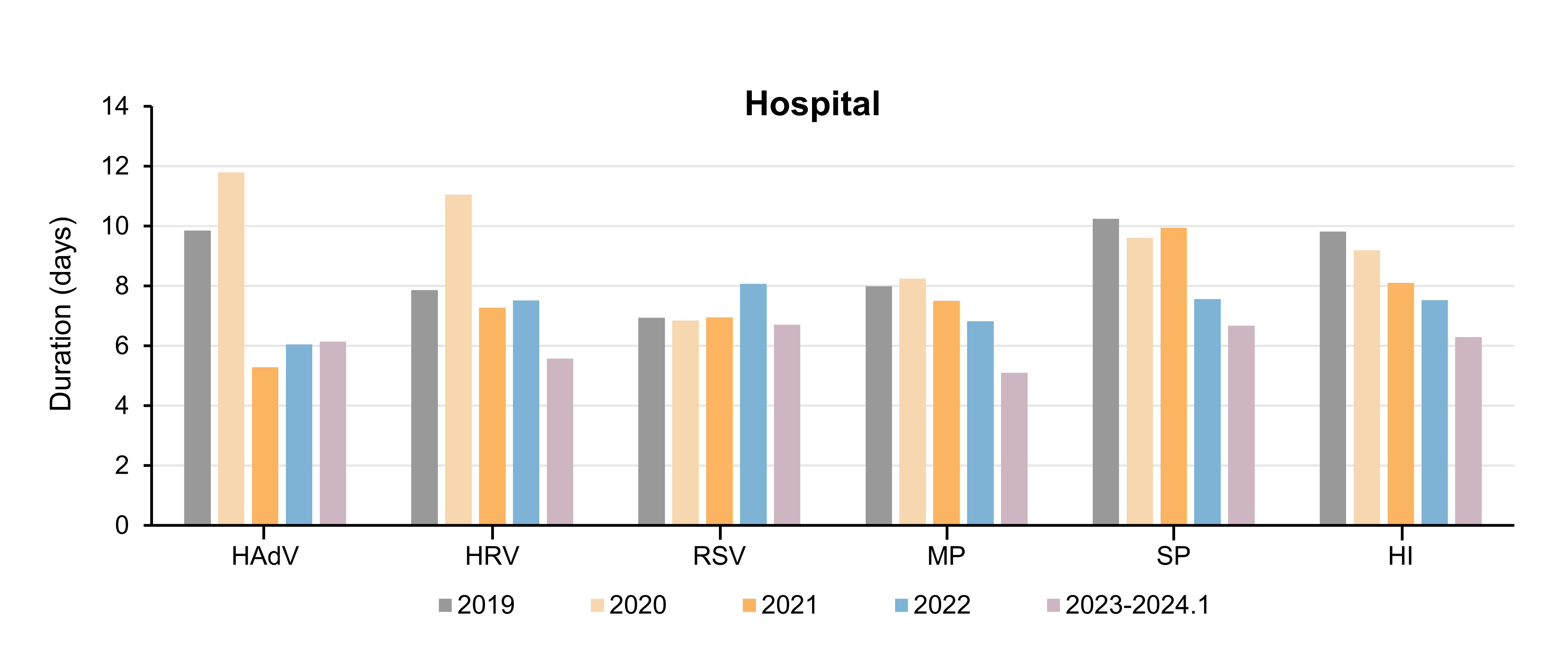


**Legend:** Duration of hospitalization for children infected with different pathogens over the past 5 years.

**Abbreviations:** human adenovirus (HAdV), human rhinoviruses (HRV), respiratory syncytial virus (RSV), *Mycoplasma pneumoniae* (MP), *Streptococcus pneumoniae* (SP), *Haemophilus influenzae* (HI).
